# Longitudinal Associations Between Energy Drink Consumption, Health, and Norm-Breaking Behavior Among Swedish Adolescents

**DOI:** 10.3389/fpubh.2021.597613

**Published:** 2021-06-11

**Authors:** Åsa Svensson, Maria Warne, Katja Gillander Gådin

**Affiliations:** ^1^Department of Health Sciences, Mid Sweden University, Östersund, Sweden; ^2^Department of Health Sciences, Mid Sweden University, Sundsvall, Sweden

**Keywords:** health behavior, risk groups, school health, support, truancy

## Abstract

**Objectives:** To describe the intake of energy drinks (EDs) among a sample of Swedish adolescents while considering health-related variables, and to investigate the longitudinal associations between ED consumption, health, and norm-breaking behavior. Longitudinal studies on this topic are scarce.

**Methods:** Questionnaire data were collected in the northern part of Sweden in 2010–2011 from 1,622 adolescents in grades 6–9. Analyses were performed using a chi-squared test and logistic regression.

**Results:** Seventy-four percent of the boys and 54% of the girls had consumed EDs (*P* < 0.001). ED consumption was associated with variables related to low health, low support levels, and norm-breaking behavior. The associations were generally stronger among the girls and the boys who consumed EDs at least once a week. ED consumption was a predictor of worse health and norm-breaking behavior 1 year later. Adjusted odds ratios ranged from 1.53 (95% CI: 1.07, 2.20; school-related stress) to 4.88 (95% CI: 2.28, 10.43; gaming-related truancy).

**Conclusions:** Health promotion activities benefit from a broad approach but could focus on girls who consume EDs and those boys with the highest consumption levels.

## Introduction

There is no evidence that limited consumption of energy drinks (EDs) is harmful, and the associated health risks are mostly related to the caffeine content (1). However, there have been reports of intoxication from excessive ED consumption (2, 3). It has been found that EDs put stress on the cardiovascular and neurological systems, which can cause arrhythmias and seizures (2, 4). In several cases, cardiovascular events have occurred as a result of ED intake in combination with alcohol or illicit drugs (3). Adolescents have reported adverse events after ED consumption, such as headache and sleeping problems (5, 6).

EDs can be described as soft drinks that contain sweeteners (whether they be sugar or artificial), caffeine, taurine, d-glucuronolactone, and B-vitamins (7, 8). Examples of other ingredients include guarana, ginseng, carnitine, creatine, gingko biloba, and different vitamins (8). The caffeine content varies, but it can be up to 400 mg/L (8). In Sweden, a can of ED usually contains 250 ml of liquid and 320 mg caffeine per liter, that is, the same amount of caffeine as a cup of coffee (7). Caffeine is a stimulant that acts on the central nervous system. Caffeine intake >400 mg could cause caffeine intoxication with serious negative health effects, especially among subgroups with cardiac or psychiatric conditions (9). However, limited amounts of caffeine are generally considered harmless, even for children and adolescents who have lower body mass and less tolerance (9).

Nevertheless, consumers of EDs have reported more adverse events compared to consumers of coffee (5). The pharmacological properties of the various herbal supplements in EDs and the possible interactions between the different agents are not fully known, and there could also be harmful effects of EDs that are not related to the caffeine content (2, 4, 9). Dietary habits in childhood and adolescence often carry forward into adulthood (10). Fortunately, health inequalities later in life can be counteracted by working to ensure that children eat healthy diets.

Adolescents' consumption of EDs is of concern not only because of the potential negative health effects of the ingredients, but also because of the associations with health risk behaviors and worse health. Associations have been found between the consumption of EDs and substance use (e.g., alcohol, cigarettes, or marijuana) (11), the use of illicit drugs (12), the consumption of other high-sugar beverages (13), and less hours of sleep (14) or late bedtime (6). One review in particular found that ED consumption was associated with mental health problems such as stress, anxiety, and depression among a majority of those included in the relevant studies (15). However, it should be noted that most of the included studies in this work used cross-sectional data, and the review itself considered null findings as well (15). Nevertheless, ED consumption among teens has also been associated with a higher body mass index (BMI) (13).

Adolescence is a time during which young individuals experience both physical and mental changes. Ultimately, during adolescence, they test the limits of what behavior is accepted in the context in which they live. This explains why breaking societal norms is most common among adolescents, and why it can be seen as a natural part of maturity (16). On the other hand, breaking societal norms has been associated with certain health problems (17) along with lower school achievement (18). Norm-breaking behavior has been described as behavior related to the use of alcohol and/or illicit drugs (19), issues with school adjustment (18), or the committing of crimes (17). In their study on students between the years of 7–9 in compulsory school, Nygren et al. (17) included questions about patterns of behavior involving breakfast, tobacco, alcohol, drugs, crime (e.g., carrying a knife as weapon), bullying, and truancy. In line with this approach, our study defined norm-breaking behavior as the transgression of laws (specifically those pertaining to tobacco, alcohol, and drug use as well as truancy) or health risk behaviors (not eating breakfast or lunch every school day).

There is a need for more studies examining the associations between ED consumption and other health and behavioral outcomes; it is also critical that the directions of the associations be considered as well. Previous studies on adolescents' ED consumption and the association with health and health-related behaviors have mainly used cross-sectional data, and thus there is a lack of longitudinal studies that have been conducted in a Scandinavian setting.

The objectives of this study were to describe the intake of EDs among a sample of Swedish adolescents in relation to their social backgrounds, levels of support, health and norm-breaking behaviors, and to investigate the longitudinal associations of ED consumption with respect to health and norm-breaking behavior.

## Methods

### Setting and Participants

The study was based on data collected between 2010 and 2011 for the project on “Youth Health Development.” This particular project was conducted in a medium-sized municipality with ~59,000 inhabitants in the northern part of Sweden. The overall aim of the project was to explore factors relevant to mental health among the youth, and to develop methods for school health promotion. The data in the present study comes from a questionnaire distributed in January of 2010 and 2011. All public and independent schools in the municipality with junior high school students (aged 12–16 years through grades 6–9) were invited to participate. One of the four independent schools and all nine of the public schools accepted the invitation. Parents and students received an informational letter articulating the voluntary nature of the study. Parents could actively decline the participation of their children. The study was reviewed and approved by Regional Ethical Review Board in Umeå, Sweden (Dnr: 09-179M).

The electronic questionnaire was built with Easy Research software and distributed to the students via their school e-mail accounts; each student received their own unique identification information. The students completed the questionnaire during school hours in a computer room. They were informed that they could decline participation at any time. The targeted resources were administered such that at least one member of the school staff could be present to make sure that all students were able to complete the questionnaire without being disturbed by others. A total of 1,622 adolescents participated in 2010, including 828 (51%) girls and 794 (49%) boys. The response rate was 77.3%. In 2011, 982 of these adolescents participated, including 530 (54%) girls and 452 (46%) boys. Grades 6–9 were evenly represented in the data.

### Measurements

Consumption frequencies of EDs was the dependent variable. The central question was as follows:

“How often do you drink EDs (e.g., Red Bull, Monster, Burn, Power King, and other similar beverages)?” The six response alternatives were: (1) “I don't drink EDs”; (2) “every day”; (3) “several times per week”; (4) “at least once a week, but not every day”; (5) “less than once a week”; and (6) “seldom.”

The independent variables included in the present study were chosen based on previous research on the associations between ED consumption and health-related behavior. We grouped the variables into the categories of social background, levels of support, health conditions, and participation in norm-breaking behavior. The included variables are described in Table 1.

**Table 1:** Overview of the included variables.

**Variable**	**Instrument or wording**	**Response choices and categorization**
**Social background**		
Not living with both parents	“With whom do you live?”	Other response alternative than “Both mother and father” = not living with both parents
Migrant background	“In which country were you born?” “In which country was your mother born?” “In which country was your father born?”	Student and/or one or two parents born in another country than Sweden = migrant background
Low personal relative affluence	“If you consider the past 3 months, have you had enough money to be able to do the same things as your friends?” This variable has been used previously to estimate the socio-economic position of adolescents (20)	“Often” or “Always” = high personal relative affluence “Never,” “Seldom,” and “Sometimes” = low personal relative affluence
**Support**		
Low parental support	“Do you usually talk about most things with your mother?” “Do you usually talk about most things with your father?”	“Always,” “Often,” “Sometimes,” “Seldom,” “Never,” “Don't have one/never see them” A sum index was calculated for the two items and the index was dichotomized based on the distribution of the answers, with the lower quartile indicating a low degree of parental support
Low peer support	“Does it happen that you are alone even if you do not want to be?” “Do you have as many friends as you want to have?” “Do you feel excluded from the peer group?”	“Always,” “Often,” “Sometimes,” “Seldom,” “Never” A sum index was calculated for the three items and the index was dichotomized based on the distribution of the answers, with the upper quartile indicating a low degree of peer support
Low teacher support	“Do you think teachers will give you help when you need it?” “Do you think your teachers would notice if you did not carry on with your school work?” “Do you think your teachers treat you fairly?” “Do your teachers praise and encourage you?”	“Always,” “Often,” “Sometimes,” “Seldom,” “Never” A sum index was calculated for the four items and the index was dichotomized based on the distribution of the answers, with the lower quartile indicating a low degree of teacher support
**Health**		
Psychosomatic problems	Hagquist (21) has described the Psychosomatic Problems Scale. The scale consists of eight items measuring difficulty in concentrating, difficulty in sleeping, headaches, stomach aches, feeling tense, having little appetite, feeling sad and feeling dizzy. The items referred to the last 6 months	“Always,” “Often,” “Sometimes,” “Seldom,” “Never” A sum index was calculated for the eight items and the index was dichotomized based on the distribution of the answers, with the upper quartile indicating high psychosomatic problems
Low quality of life	The adapted Cantril's Ladder (22) was used, where the respondent is asked to rate their life satisfaction on a scale ranging from the worst possible life (0) to the best possible life (10), on a picture of a ladder.	6–10 = High quality of life 0–5 = Low quality of life This cut-off has been used in Health Behaviors in School-aged Children (23)
Deliberate self-harm	“Have you during the past year…? • Thought about harming yourself by e.g., cutting, scratching, burning or puncturing yourself? • Harmed yourself by e.g. cutting, scratching, burning or puncturing yourself? • Harmed yourself so much that you needed to seek medical care? • Taken an overdose of medicine in order harm to yourself?”	Yes to any of the questions = deliberate self-harm
Negative body image	“Are you happy with.? • The way you look • Your body • Your height • Your weight”	“Always,” “Often,” “Sometimes,” “Seldom,” “Never” A sum index was calculated for the four items and the index was dichotomized based on the distribution of the answers, with the lower quartile indicating a negative body image
School-related stress	“I you consider the last 6 months, have you felt stressed about school work?”	“Always” or “Often” = School-related stress “Sometimes,” “Seldom,” and “Never” = No school-related stress
Not enough sleep	“Do you get enough sleep on school-nights?”	“Always” or “Often” = Enough sleep “Sometimes,” “Seldom,” and “Never” = Not enough sleep
**Norm-breaking behavior**		
General truancy	“How many times during the last semester have you played truant?”	Ever
Gaming-related truancy	“Have you stayed at home from school to play computer games during the last semester?”	Ever
Alcohol use	“Have you ever used alcohol?”	Yes/No
Tobacco use	“Have you ever smoked cigarettes?” “Have you ever used snuff?”	Yes to any of the questions = Tobacco use
Not breakfast every school day	“What is it usually like during school days; how often do you eat breakfast?”	Other answer than every day = Not breakfast every school day
Not lunch every school day	“What is it usually like during school days; how often do you eat lunch?”	Other answer than every day = Not lunch every school day

### Statistical Analysis

Statistical analysis was performed with IBM SPSS Statistics version 22, and *p* ≤ 0.05 were considered significant. For the cross-sectional analyses, data from 2010 was used. Descriptive data was presented as frequencies and percentage proportions. In order to investigate the cross-sectional associations between consumption of EDs (“never” vs. “ever,” and, in an additional analysis for boys, “never” vs. “once a week or more”) and social background, levels of support, health conditions, and participation in norm-breaking behavior, Pearson's chi-squared test was used. Longitudinal analyses were conducted for data collected in 2010 and 2011 using logistic regression models for both the entire sample and separately for boys and girls. The following example explains the procedure of the longitudinal analysis: We investigated alcohol consumption (“never” vs. “ever”) in 2011 as a function of ED consumption (“never” vs. “ever”) at the baseline in 2010 for the sub-sample who had not consumed alcohol. Additionally, models were performed following the same rationale but with the variables reversed, i.e., ED consumption in 2011 was investigated as a function of alcohol consumed at the baseline for the sub-sample that had not consumed EDs. This method has been used previously (24). Logistic regression models were similarly conducted to investigate the longitudinal associations between ED consumption and the presence of: (1) significant psychosomatic problems, low quality of life, deliberate self-harm, negative body image, school-related stress, insufficient sleep, general truancy, gaming-related truancy, tobacco use, not eating breakfast every school day, and not eating lunch every school day. The categorization of response alternatives are described in more detail in Table 1. Grade (6, 7, 8, or 9) and personal relative affluence (low vs. other) were included as covariates. For boys, additional analyses were conducted in a similar way albeit with a variable ED consumption of once a week or more since we wanted to investigate the boys with the highest levels of consumption. Few girls consumed EDs, and therefore “ever” represented the girls with the highest levels of consumption. Logistic regression models with variable categories and *n* < 10 are not presented.

## Results

### Descriptive Statistics

Self-reported consumption of EDs in 2010 and 2011 is presented in Table 2. Frequent consumption of EDs was more common among boys than girls (*p* < 0.001 in 2010 and 2011), and the majority of both boys and girls either never or seldom consumed EDs (Table 2).

**Table 2:** Consumption of energy drinks among Swedish boys and girls in grades 6–9 in 2010 and 2011 (only those participating in 2010).

	**Boys**		**Girls**		**All**	
	***n***	**%**	***n***	**%**	***n***	**%**
**2010**	***n*** **=** **730**		***n*** **=** **795**		***n*** **=** **1,525**	
Never	193	26.4	367	46.2	560	36.7
Seldom	262	35.9	298	37.5	560	36.7
Less than once a week	107	14.7	70	8.8	177	11.6
At least once a week but not every day	76	10.4	22	2.8	98	6.4
Several times per week	62	8.5	28	3.5	90	5.9
Every day	30	4.1	10	1.3	40	2.6
**2011**	***n*** **=** **404**		***n*** **=** **504**		***n*** **=** **908**	
Never	94	23.3	201	39.9	295	32.5
Seldom	167	41.3	215	42.5	382	42.1
Less than once a week	70	17.3	43	8.5	113	12.4
At least once a week but not every day	27	6.7	30	6.0	57	6.3
Several times per week	27	6.7	13	2.6	40	4.4
Every day	19	4.7	2		21	2.3

*Results are presented as n (%)*.

Descriptive statistics of the variables representing social background, levels of support, health conditions, and participation in norm-breaking behavior in 2010 are presented in Table 3.

**Table 3:** Characteristics (percentages) of girls and boys who reported to never or ever consume energy drinks (EDs), and boys who reported to consume EDs once a week or more, in 2010.

	**Girls**				**Boys**					
	**All, %**	**Never EDs, %**	**Ever EDs, %**	***P*-value**	**All, %**	**Never EDs, %**	**Ever EDs, %**	***P*-value**	**EDs once a week or more, %**	***P*-value**
**Social background**										
Not living with both parents	38.4	**29.5**	**47.1**	**<0.001**	35.8	35.9	35.2	0.85	44.0	0.12
Migrant background	14.8	15.3	12.9	0.33	12.3	9.4	12.4	0.27	**16.9**	**0.04**
Low personal relative affluence	20.7	**16.2**	**24.5**	**0.004**	16.0	12.5	16.1	0.23	**20.8**	**0.03**
**Support**										
Low parental support	23.2	**18.2**	**27.7**	**0.002**	27.3	**20.9**	**29.4**	**0.03**	**37.0**	**0.001**
Low peer support	30.7	30.3	30.4	0.97	27.6	28.7	26.9	0.63	27.7	0.83
Low teacher support	33.4	**28.0**	**38.2**	**0.003**	32.1	**22.4**	**35.7**	**0.001**	**52.7**	**<0.001**
**Health**										
High psychosomatic problems	38.7	**28.1**	**48.6**	**<0.001**	15.8	**8.9**	**16.6**	**0.01**	**25.5**	**<0.001**
Low quality of life	30.3	**26.4**	**32.9**	**0.04**	22.3	**16.2**	**24.9**	**0.01**	**28.7**	**0.004**
Deliberate self-harm, ever	28.9	**19.9**	**36.5**	**<0.001**	17.6	13.8	18.9	0.11	20.5	0.09
Negative body image	43.3	**33.9**	**50.9**	**<0.001**	19.0	**10.6**	**22.1**	**0.001**	**28.8**	**<0.001**
School-related stress	40.5	**34.2**	**46.0**	**0.001**	26.7	**20.0**	**27.4**	**0.04**	**29.7**	**0.03**
Not enough sleep	52.8	**42.2**	**62.4**	**<0.001**	48.6	**38.1**	**52.3**	**0.001**	**67.1**	**<0.001**
**Norm-breaking behavior**										
General truancy	23.4	**12.3**	**32.2**	**<0.001**	22.3	**8.4**	**26.6**	**0.001**	**50.3**	**<0.001**
Gaming-related truancy	7.9	**3.8**	**11.5**	**<0.001**	20.4	**10.9**	**23.8**	**<0.001**	**42.2**	**<0.001**
Alcohol use, ever	55.5	**39.3**	**69.6**	**<0.001**	56.1	**33.3**	**64.1**	**<0.001**	**81.4**	**<0.001**
Tobacco use, ever	35.7	**18.9**	**50.2**	**<0.001**	31.7	**8.9**	**39.8**	**<0.001**	**59.6**	**<0.001**
Not breakfast every school day	36.8	**20.9**	**50.4**	**<0.001**	25.3	**14.7**	**29.2**	**<0.001**	**40.2**	**<0.001**
Not lunch every school day	47.0	**36.0**	**64.0**	**<0.001**	36.5	**26.8**	**40.1**	**0.001**	**54.6**	**<0.001**

*Differences at the significance level <0.05 derived from the Pearson chi-squared test are in bold. N varied between 1,388 (732 girls and 656 boys) and 1,522 (793 girls and 729 boys), and 323 and 360 boys, depending on the variable*.

### Cross-Sectional Associations

Several variables were associated with ED consumption (Table 3). Among the girls, consumption of EDs was more common among those (1) not living with both parents, and (2) reporting low personal relative affluence. Furthermore, the girls who reported low levels of parental and teacher support were more likely to consume EDs. All investigated health-related and norm-breaking behavior variables were associated with ED consumption among the girls. For the boys, low levels of parental and teacher support were associated with ED consumption, as was the case with all of the norm-breaking behavior variables and health-related variables except for deliberate self-harm. Additional analyses were performed for the boys by comparing characteristics of those who reported to never consume EDs and those who reported to consume EDs once a week or more. This analysis showed stronger associations between ED consumption and low levels of support, worse health, and increased rates of norm-breaking behavior (Table 3). Furthermore, ED consumption was associated with a migrant background and low personal relative affluence in this analysis (Table 3).

### Longitudinal Associations

We tested for longitudinal associations between ED consumption and health-related and norm-breaking behaviors. Controlling the analyses for grade and personal relative affluence did not substantially alter the results, and the adjusted odds ratio (OR) values are presented in Table 4. The analyses showed positive associations between ED consumption in 2010 and school-related stress, general truancy, gaming-related truancy, tobacco use, and not eating breakfast every school day in 2011 among the subsamples that did not report such issues in 2010 (Table 4). The OR values were higher when analyzing the sample of boys who reported consuming EDs at least once per week in comparison to the larger sample of boys who reported to have ever consumed EDs (Table 4). For both the subset of girls and the total sample, associations were also observed among ED consumption in 2010 and deliberate self-harm, not getting enough sleep, and alcohol consumption in 2011 (Table 4). For boys, an association was observed between ED consumption in 2010 and significant psychosomatic problems in 2011 among the sample of those who consumed EDs at least once per week (Table 4).

**Table 4:** Results of logistic regression models adjusted for grade (6–9) and personal relative affluence in 2010.

	**All**	**Girls**	**Boys**	**Boys**
	**Ever EDs**	**Ever EDs**	**Ever EDs**	**EDs once a week or more**
	**OR [95% CI]**	**OR [95% CI]**	**OR [95% CI]**	**OR [95% CI]**
**Health**				
EDs 2010 → High psychosomatic problems 2011	1.25 [0.78, 2.00]	1.43 [0.79, 2.58]	1.76 [0.73, 4.23]	**3.44** [**1.24, 9.53**]
High psychosomatic problems 2010 → EDs 2011	1.17 [0.65, 2.10]	1.64 [0.82, 3.26]	0.66 [0.18, 2.42]	–^a^
EDs 2010 → Low quality of life 2011	1.27 [0.84, 1.91]	1.24 [0.76, 2.01]	1.82 [0.86, 3.85]	1.38 [0.53, 3.62]
Low quality of life 2010 → EDs 2011	0.94 [0.51, 1.73]	0.92 [0.44, 1.92]	1.22 [0.38, 3.88]	–^a^
EDs 2010 → Deliberate self-harm 2011	**1.68** [**1.06, 2.66**]	**2.49** [**1.38, 4.48**]	1.10 [0.52, 2.34]	2.41 [1.00, 5.82]^d^
Deliberate self-harm 2010 → EDs 2011	1.57 [0.87, 2.84]	1.48 [0.73, 3.00]	2.67 [0.78, 9.15]	–^a^
EDs 2010 → Negative body image 2011	1.09 [0.69, 1.72]	1.57 [0.90, 2.77]	1.12 [0.47, 2.69]	1.02 [0.29, 3.65]
Negative body image 2010 → EDs 2011	0.55 [0.29, 1.02]	0.70 [0.34, 1.46]	0.50 [0.12, 2.08]	–^a^
EDs 2010 → School-related stress 2011	**1.53** [**1.07, 2.20**]	**1.74** [**1.09, 2.78**]	1.88 [0.98, 3.59]^c^	**2.93** [**1.31, 6.55**]
School-related stress 2010 → EDs 2011	1.51 [0.88, 2.60]	**2.06** [**1.07, 3.99]^b^**	1.10 [0.35, 3.47]	–^a^
EDs 2010 → Not enough sleep 2011	**1.54** [**1.02, 2.32**]	**2.39** [**1.39, 4.09**]	1.02 [0.53, 1.99]	0.74 [0.22, 2.51]
Not enough sleep 2010 → EDs 2011	**1.71** [**1.05, 2.78**]	1.56 [0.84, 2.90]	2.04 [0.88, 4.73]	–^a^
**Norm-breaking behavior**				
EDs 2010 → General truancy 2011	**3.03** [**1.05, 3.02**]	**3.67** [**1.96, 6.87**]	**2.40** [**1.06, 5.40**]	**6.03** [**2.28, 15.93**]
General truancy 2010 → EDs 2011	1.63 [0.67, 3.99]	1.19 [0.40, 3.52]	–^a^	–^a^
EDs 2010 → Gaming-related truancy 2011	**4.88** [**2.28, 10.43**]	**4.40** [**1.25, 15.52**]	**3.63** [**1.38, 9.58**]	**11.21** [**3.79, 33.12**]
Gaming-related truancy 2010 → EDs 2011	1.97 [0.79, 4.93]	–^a^	1.27 [0.37, 4.39]	–^a^
EDs 2010 → Alcohol use, ever 2011	**1.88** [**1.22, 2.88**]	**2.38** [**1.33, 4.27**]	1.57 [0.80, 3.10]	2.03 [0.52, 7.97]
Alcohol use, ever 2010 → EDs 2011	1.39 [0.85, 2.26]	1.54 [0.82, 2.90]	1.49 [0.63, 3.54]	–^a^
EDs 2010 → Tobacco use, ever 2011	**3.17** [**1.95, 5.17**]	**3.20** [**1.77, 5.79**]	**4.82** [**1.80, 12.87**]	**7.60** [**2.23, 25.91**]
Tobacco use, ever 2010 → EDs 2011	1.47 [0.73, 2.97]	1.82 [0.80, 4.14]	–^a^	–^a^
EDs 2010 → Not breakfast every school day 2011	**1.94** [**1.26, 2.99**]	**2.29** [**1.31, 4.01**]	2.19 [1.00, 4.80]^d^	**4.56** [**1.66, 12.53**]
Not breakfast every school day 2010 → EDs 2011	**2.04** [**1.10, 3.79**]	2.03 [0.95, 4.31]	2.44 [0.77, 7.73]	–^a^
EDs 2010 → Not lunch every school day 2011	1.33 [0.89, 1.99]	1.38 [0.78, 2.44]	1.37 [0.74, 2.54]	2.06 [0.83, 5.09]
Not lunch every school day 2010 → EDs 2011	1.15 [0.71, 1.87]	1.27 [0.69, 2.31]	1.14 [0.48, 2.68]	–^a^

*N varied between 296 (98 boys and 198 girls) and 757 (307 boys and 450 girls) depending on the analyzed variable. EDs, Energy drinks*.

*Statistically significant results are in bold*.

a *The analysis included too few participants to be performed (n < 10 for the dependent and/or independent variable)*.

b *Not statistically significant when unadjusted*.

c *Statistically significant when unadjusted*.

d *P = 0.996*.

Among those who did not consume EDs in 2010, the variables that predicted ED consumption in 2011 were school-related stress, not getting enough sleep, and not eating breakfast every school day in 2010 (Table 4). Except for school-related stress, which was seen only among girls, these results were observed among those in the total sample.

## Discussion

In the present study, boys consumed EDs more frequently than girls, although it is important to note that most of the participants either seldom or never consumed EDs. Consumption of EDs was positively associated with a majority of the analyzed variables of low levels of support, worse health, and more frequent participation in norm-breaking behaviors. The longitudinal analyses showed that, above all, ED consumption preceded both norm-breaking behavior and worse health 1 year later. The associations were generally stronger for the girls than the boys, and especially the boys with the highest levels of ED consumption.

The consumption of EDs in the present study is not comparable with the levels reported by a recent Swedish national dietary survey (25) since the latter investigated amounts whereas the present study relied on frequencies of consumption. In the national survey, administered to students in grades 5, 8, and year 2 of upper secondary school, 5% of both boys and girls consumed EDs during the 2-day data collection period (25). However, a higher intake among boys as compared to girls was seen with respect to the larger amounts of ED that were consumed (12 and 9 g per day, respectively) (25). The same national survey also collected data from youth not attending school, and in this smaller sample, 22% of boys and 9% of girls consumed EDs. A larger intake among boys has also been shown in other studies (14, 26, 27). A report from the European Food Safety Authority showed that among adolescents between 10 and 18 years of age in the European Union, ~68% were consumers of EDs (at least once during the last year) on average in the 16 surveyed countries (8).

This study used a variable for personal relative affluence to categorize adolescents' socio-economic status. This variable was associated with ED consumption among girls and the boys that consumed EDs once a week or more. This is in line with reports on differences in diet quality that result from health inequalities (28). Furthermore, previous studies on differences in ED consumption based on socio-economic have revealed both associations (26) as well as no associations (14).

In the present study, ED consumption was associated with worse health (e.g., worse mental health, less sleep, and more stress). Concerns about this type of associations, as well as concerns about ED consumption and health risk behaviors (such as smoking and drinking alcohol) among adolescents have been raised previously (4, 11, 15, 29). Thus, the results of the present study strengthen the evidence for these associations.

### Implications for Policy and Practice

ED consumption has been shown to have longitudinal associations with worse health and increased participation in norm-breaking behavior in the present study among both girls and boys. One year later, the consumption of EDs was found to be a predictor of deliberate self-harm, school-related stress, not getting enough sleep, general and gaming-related truancy, having used alcohol and tobacco, and not eating breakfast every day, but for most variables, there was no such relationship the other way round. In other words, ED consumption seems to occur before other health risk behaviors, and thus it can be construed as an indicator of worse health and increased participation in norm-breaking behavior later on. Based on the results of this study sample, ED consumption can be interpreted as a red flag that predicts other health risk behaviors such as alcohol consumption and the use of tobacco. The reason why ED consumption is prior to other behaviors could be due to the fact that such drinks are simply easier to get a hold of. In Sweden, there is an age limit to buy alcohol and tobacco, but not EDs. There have been initiatives to make retailers restrict the sale of EDs to children and adolescents by encouraging an age limit. However, several major supermarkets still do not have an age limit for EDs. Furthermore, the consumption of EDs among the young is generally more socially acceptable than the consumption of alcohol and tobacco. Nevertheless, such differences could also be a function of levels of discouragement by parents and other adults, and therefore consuming such substances is simply exciting. Peer support was not associated with ED consumption in the present study, although low levels of parental and teacher support was. It is possible, therefore, that ED is used as a “pause drink” when it is accepted by those in the peer group and other options such as alcohol are unavailable. Costa et al. (30) found that EDs were consumed by adolescents as an alternative to other drinks to provide energy and foster interaction in social contexts. Sensation-seeking has also been associated with ED consumption (11). Since ED consumption can be the first step toward further norm-breaking behaviors, an age limit is recommended to prevent further negative behaviors. In fact, students have suggested this approach themselves in a study from the United Kingdom (31). Ultimately, an age limit would send the message that EDs are not intended for children and young people (31).

It is widely known that health risk behaviors among adolescents cluster (32). Distal determinants are probably responsible for the negative patterns of behavior, including the consumption of EDs among the adolescents surveyed for the present study. Since adolescence is formative, when it comes to lifestyle, it is important to promote healthy behaviors during this critical period. An environment that supports healthy behaviors irrespective of socio-economic status is especially important for adolescents. If attention is given to the differences in health behaviors among this group, socio-economic differences in adulthood can potentially be counteracted. With this objective in mind, the “whole school” approach is a means by which adults can work broadly to ensure the health of all students (33). For example, schools could aim to promote health literacy among the students, thereby building competencies that aid healthy dietary choices (34). Schools could also work to ensure that healthy alternatives to EDs are available in school cafeterias. Furthermore, supportive relations with parents and teachers are associated with students' improved health (35). Among the girls and boys surveyed in the present study, 23–33% reported low levels of support from parents and teachers, which shows that there is certainly some room for improvement. In general, adolescents consuming EDs should be observed when it comes to the issue of monitoring health risk behaviors, and in this respect, exchanges with school nurses could be used as an opportunity to discuss this issue (36). However, it has been shown that students who engage in health risk behaviors such as smoking and drinking are less likely to reflect on the content of the dialogue and follow the nurse's advice than students who do not participate in such activities (37).

Although the consumption of EDs was found to be more common among boys than girls, the stronger association for girls with most of the investigated variables indicates that girls who consume EDs are a more specific risk group. For boys, the associations were stronger when analyzing those who consumed EDs more frequently than once a week. Therefore, to identify boys most at risk for worse health and increased rates of norm-breaking behavior, one might need to use a higher limit of consumption for screening.

### Methodological Considerations

The strengths of the present study include the longitudinal school-based design, the relatively large sample size, and the low drop-out rate. However, some limitations should be kept in mind when interpreting the results. First, the questionnaire was not designed to study ED consumption specifically. Therefore, only one question about this core variable was included, and the time-period that the question referred to was not specified. Accordingly, many of the participants replied “seldom” to the question of ED consumption. However, the analysis used broad categories of ED consumption (“never” vs. “ever” and “never” vs. “once a week or more”), and these alternatives probably attenuated the effects of any recall bias. Even these broad categories of consumption showed strong associations with many of the investigated variables. Furthermore, even though the sample was large to begin with, there were fewer responses to some of the variables and some response categories. Because of this, the sample size for some of the longitudinal analyses might have been too small to detect statistically significant associations. Moreover, the high number of hypothesis tests in this study increased the risk of false positives. The questionnaire contained questions about issues that could be perceived as sensitive (such as alcohol consumption). Previous research have found that asking adolescents about risky behavior does not increase the behavior (38). At the end of the questionnaire, the student were asked to indicate if they had been affected by the questions and wished to be contacted by the school nurse or counselor.

## Conclusions

To summarize, the results showed that adolescents' consumption of EDs was associated with worse health and increased participation in norm-breaking behaviors both concurrently as well as 1 year later. The associations were generally stronger for girls than boys, suggesting that the relatively smaller group of girls who consume EDs is more at risk. More specifically, stronger associations were observed for boys when including those who consumed EDs once a week or more often. Ultimately, it is apparent that young consumers of EDs are important to target with health promotion activities. Health promotion activities should have a broad approach, but there is also reason to focus especially on girls who consume EDs and the boys with the highest the ED consumption rates. The underlying risk factors of ED consumption and its health effects need to be studied further. Future research should focus on disentangling the relationships between ED consumption, the participation in health-related behaviors, and health conditions, as well as investigating any common determinants. Therefore, study designs should be longitudinal and include enough participants to be able to control for several confounders to ensure that there is enough power to detect the relevant associations.

## Data Availability Statement

The datasets presented in this article are not readily available because of ethical reasons. Requests to access the datasets should be directed to Katja Gillander Gådin, katja.gillandergadin@miun.se.

## Ethics Statement

The studies involving human participants were reviewed and approved by Regional Ethical Review Board in Umeå, Sweden (Dnr: 09-179M). Written informed consent from the participants' legal guardian/next of kin was not required to participate in this study in accordance with the national legislation and the institutional requirements.

## Author Contributions

ÅS analyzed the data and drafted the manuscript. MW and KG acquired the data and critically revised the manuscript for important intellectual content. All authors contributed to the design of the study and approved the final version and take responsibility for the content.

## Conflict of Interest

The authors declare that the research was conducted in the absence of any commercial or financial relationships that could be construed as a potential conflict of interest.
